# Effect of six different autorefractor designs on the precision and accuracy of refractive error measurement

**DOI:** 10.1371/journal.pone.0278269

**Published:** 2022-11-28

**Authors:** Abinaya Priya Venkataraman, Rune Brautaset, Alberto Domínguez-Vicent

**Affiliations:** Division of Eye and Vision, Department of Clinical Neuroscience, Karolinska Institute, Stockholm, Sweden; The Ohio State University, UNITED STATES

## Abstract

**Purpose:**

To evaluate the precision of objective refraction measurements with six different autorefractors that have different designs and measurement principles and to compare the objective refraction values with the subjective refraction.

**Method:**

Objective refraction of 55 participants was measured using six autorefractors with different designs. The instrument features mainly varied in terms of measurement principles, inbuilt fogging, open or closed view, and handheld or stationary designs. Two repeated measurements of objective refraction were performed with each autorefractor. The objective refractions from the six autorefractors were compared with the standard subjective refraction. The repeatability limit and Bland-Altman were used to describe the precision and accuracy of each autorefractor, respectively. The analysis was done using the spherical component of the refraction and the power-vector components, spherical equivalent (M), and cylindrical vectors.

**Results:**

The repeatability of all autorefractors was within 1.00 and 0.35D for measuring the M and both cylindrical components, respectively. Inbuilt fogging was the common feature of the instruments that showed better repeatability. Compared to subjective refraction, the mean difference for sphere and M was below +0.50D, and it was close to zero for the cylindrical components. The instruments that had inbuilt fogging showed narrower limit of agreement. When combined with fogging, the open field refractors showed better precision and accuracy.

**Conclusions:**

The inbuilt fogging is the most important feature followed by the open view in determining the precision and accuracy of the autorefractor values.

## 1. Introduction

The prevalence of refractive error is increasing globally [1–3] and hence more people are dependent on spectacles or contact lenses for sharp vision. Subjective or manifest refraction is considered the gold-standard to determine the optimal optical correction needed as it takes into account both optical and neurological factors. Less myopic values are obtained when subjective refraction is performed with cycloplegic agents that paralyze the ciliary muscles and arrest accommodation [4–7]. This effect has been shown to be limited in subjects older than 20 years old, and it has been suggested that cycloplegic refraction is of less clinical relevance [6, 7]. Autorefraction is considered a reliable and fast method to measure refraction objectively in a general population, and it is being used to provide reliable preliminary start point for the subjective refraction. Since 1970 [8], there has been a rapid development of clinically available autorefractors. The recent autorefractors are designed to provide refraction values as close as the subjective refraction. Nevertheless, the autorefraction has not been able to completely replace subjective refraction due to several factors including accommodation and blur sensitivity which are taken into account during subjective refraction.

The autorefractors vary mainly in terms of the optical design and measurement principles. Depending on the instrument design, the objective refraction can be measured monocularly or binocularly, with or without open field viewing, with or without fogging, and through central or the whole pupil area. The objective refraction is evaluated with several different measurement principles including wavefront analysis, Scheiner disc principle, best focus principle, ray-deflection, and photo refraction [9]. The pupil zone used for refraction measurement is also different in these methods. Previous studies have shown the objective refraction measurements obtained with different autorefractors vary, and it is suggested that this variation could be due to the measurement principle and design of each instrument [10–14]. The autorefractors are available as tabletop or handheld, and the clinical studies have shown that on average the differences among these two types of autorefractors are not clinically significant [12, 15].

The accuracy of the autorefractors is assessed by comparing the objective refraction values with the subjective refraction [16]. It is known that the objective and subjective refraction values differ, mainly due to the instrument measurement principle and design. For example, autorefractors with an inbuilt fogging system measure provide less myopic or more hyperopic values compared to the designs without fogging [13, 17]. Closed-field autorefractors can induce instrument myopia [14]. Another source of disagreement between subjective and objective refraction values could be that the former considers both optical and neurological factors, and the latter considers only the optical factors. The reliability of an autorefractor can be described in terms of precision and accuracy. The design and measurement principle of an autorefractor can influence either the precision or accuracy or both. Hence, it is important to evaluate which combination (fogging/no fogging, monocular/binocular, open field/closed field view, handheld/tabletop) provides the most reliable refraction measurement.

In the present study, the repeatability of the objective refraction measurements with six different autorefractors that have different designs and measurement principles was evaluated to address the measurement precision. We also compared the objective refraction values of each autorefractor with the subjective refraction to assess the accuracy of each autorefractor.

## 2. Methods and materials

### 2.1 Subjects

A total of 55 participants (mean age of 26.05 ± 3.98 years; 14 men and 41 women) were included in the present study. Only data from one eye from each participant was analyzed in order not to artificially reduce the confidence interval around the limits of agreement [18].

The study was approved by the Regional Ethical Committee (Swedish Ethical Review Authority), and the study procedures adhered to the tenets of the Declaration of Helsinki. Each participant signed the informed consent after explaining the purpose, nature, and possible consequences of the study.

The inclusion criteria to participate in this study were no history of refractive surgery procedure or history of ocular disease; no ocular dysfunctions that could affect the refraction; best corrected visual acuity of 0.0 logMAR or better; intraocular pressure below 21 mmHg (measured with non-contact tonometer); no pregnancy or lactation; and no use of any systemic or ocular medication that could have any impact on the refraction.

### 2.2 Instrumentation

Six different autorefractors were used in this study: the WaveAnalyzer 700 (Essilor, France), the Eye Refract (Visionix, France), the NVision-K 5001 (Shin-Nippon, Japan), the PlusoptiX A12C (Plusoptix GmbH, Germany), the TONOREF™ III (Nidek, Japan), and the Handheld ref/keratometer HandyRef-K (Nidek, Japan). These instruments have different measurement principles and designs and were used to measure the objective refraction of each participant. Table 1 summarizes the main features of these autorefractors.

**Table 1:** Summary of the features of each autorefractor.

Autorefractor	Measurement principle	Fogging	Open/Binocular view	Simultaneous binocular measurement	Tabletop/Handheld
**Visionix Eye Refract**	Wavefront	Yes	Yes	Yes	Tabletop
**WaveAnalyzer 700**	Wavefront	Yes	No	No	Tabletop
**Nidek TONOREF™ III**	Retinal image size	Yes	No	No	Tabletop
**Handheld ref/keratometer HandyRef-K**	Retinal image size	Yes	No	No	Handheld
**PlusoptiX A12C**	Photorefraction	No	Yes	Yes	Handheld
**Shin-Nippon NVision-K 5001**	Retinal image size	No	Yes	No	Tabletop

The **Visionix Eye Refract** (VX) is a binocular and tabletop refractor that provides a semi open field view, and it measures the objective refraction with fogging and simultaneously on both eyes. This autorefractor combines a digital phoropter with a dual Hartman-Shack sensor. In this study, the participants fixated at a scenery chart displayed on a digital screen at 4.0m from the instrument.

The **WaveAnalyzer 700** (WA) combines a Placido disc ring, a Scheimpflug camera, and a Hartmann-Shack sensor to measure anterior segment parameters and the objective refraction. This is a tabletop and closed field autorefractor that measures the objective refraction monocularly with fogging technique. During the measurements, the subject fixated at a scenery chart inside the instrument.

The **Nidek TONOREF™ III** (NIII) provides automatic measurements of the objective refraction, keratometer, and non-contact tonometry and pachymetry. This tabletop autorefractor measures the objective refraction monocularly with fogging while the subject is fixating at a scenery chart inside the instrument. As a standard setting during each refraction measurement, 3 consecutive measurements are performed in rapid succession and the average values are reported. The subject’s astigmatism is corrected using built-in cylindrical lenses before proceeding with the fogging.

The **Handheld ref/keratometer HandyRef-K** (NH) measures the objective refraction monocularly with fogging. During the objective refraction measurement, the subject was looking monocularly to a scenery view that it was placed inside the instrument. As a standard setting during each refraction measurement, 3 consecutive measurements are performed in rapid succession and the average values are reported.

The **PlusoptiX A12C** (PO) is a handheld autorefractor that measures the objective refraction simultaneously on both eyes using photorefraction principle. The illumination system contains 54 infrared LEDs distributed in a hexagonal pattern. During the measurement, the examiner held the instrument at 1.0 m distance from the subject, and the subject was instructed to look at the nose of a built-in smiley face, which is aligned with the center of the illumination system.

The **Shin-Nippon NVision-K 500** (SN) measures the objective refraction monocularly without fogging. This tabletop instrument is open field, and during the measurement, the participants fixated binocularly to a Maltese Cross placed at 4.0m from the instrument. As a standard setting during each refraction measurement, 3 consecutive measurements are performed in rapid succession and the average values are reported.

### 2.3 Measurements

The refraction of each participant was measured with all six autorefractors, and with a standard subjective refraction method.

#### 2.3.1 Autorefraction

For each participant, the instrument order was randomized. In total, all participants underwent two repeated measurements with each instrument. After the first measurements with each autorefractor the subjects were instructed to sit back for 2 minutes and then the second measurement was performed. All measurements were taken on the same day on one randomly chosen eye. Each individual measurement took less than 1 minute.

#### 2.3.2 Subjective refraction

The subjective refraction was measured by the same examiner. The starting value for the subjective refraction was chosen from the objective refraction measured with the WaveAnalyzer 700. The subjective refraction was performed under photopic conditions, with the same illumination level for all subjects, and no cycloplegic agents were used. The endpoint for the subjective refraction was set as the maximum positive/minimum negative spherical value that gives the maximum visual acuity. A conventional fogging method was used, and the Jackson Cross cylinder technique was used to refine the cylinder. From the binocular refraction performed, only the value from the eye measured on the autorefractors was included in the analysis.

### 2.4 Data analysis

The manifest and objective refractions were measured using the spherocylindrical notation, and for the analysis, these values were converted into power-vector notation using the following equations [19]:

$$M=S+\frac{C}{2}$$

$$J0=-\frac{C}{2}∙\cos\;2∙\alpha$$

$$J45=-\frac{C}{2}∙\sin\;2∙\alpha$$

In these equations, M represents the spherical equivalent, J0 and J45 represent the cylindrical vectors, S, C and α represent the spherical power, the negative value of the cylindrical power, and the cylinder axis respectively.

Descriptive statistics were used to summarize the baseline demographics of the results obtained from each measurement and autorefractor. The repeatability of each instrument was described in terms of within subject standard deviation (Sw) using a one-way analysis of variance (ANOVA) with the subjects as a factor [18]. The repeatability limit was calculated as $1.96∙\sqrt{2}∙S_{w}$, and it represents the expected limits that 95% of the measurements should be within. The agreement between the objective and subjective refraction was assessed using a Bland-Altman analysis [20].

The normality distribution of the power vector components was tested using Q-Q plots. As the data was not normally distributed, the Friedman repeated-measurements ANOVA was used to compare M, J0 and J45, between the different refraction methods. When there were statistically significant differences, the Tukey test was used to identify whether the differences among the autorefractors and subjective refraction were statistically significant.

Since only one measurement was taken during the subjective refraction, and two measurements were taken on each autorefractor, the average of both measurements were used for the Bland-Altman and ANOVA analyses. The statistical significance limit was set to a p-value < 0.05.

## 3. Results

### 3.1 Repeatability

The average refractive values obtained with each measurement and autorefractor are shown in Table 2. The subjects’ refraction ranged from -5.25 to +4.25D based on the M value for the subjective refraction. The differences among the two measurements taken with the same autorefractor were on average smaller than 0.15D for each refractive component, and these differences were not statistically significant (p > 0.05).

**Table 2:** Refractive outcomes obtained with each autorefractor.

	SPH		M		J0		J45	
Measurements	1	2	1	2	1	2	1	2
**VX**	-0.62±1.60	-0.57±1.62	-0.88±1.64	-0.81±1.66	0.03±0.23	0.04±0.21	0.01±0.22	0.01±0.23
**WA**	-0.77±1.55	-0.65±1.60	-1.05±1.61	-0.90±1.67	0.02±0.25	0.04±0.25	0.02±0.26	0.02±0.25
**NIII**	-0.66±1.65	-0.62±1.68	-0.94±1.68	-0.88±1.71	0.01±0.24	0.03±0.29	0.07±0.27	0.03±0.22
**NH**	-0.80±1.45	-0.76±1.39	-1.05±1.50	-1.01±1.43	0.06±0.24	0.06±0.24	0.03±0.24	0.02±0.24
**PO**	-1.07±1.62	-1.01±1.70	-1.26±1.69	-1.21±1.77	0.06±0.19	0.08±0.21	0.01±0.15	0.02±0.15
**SN**	-0.41±1.47	-0.42±1.47	-0.67±1.50	-0.69±1.50	0.00±0.24	0.00±0.23	-0.07±0.20	-0.07±0.21
**SR**	-0.58±1.67	N/A	-0.83±1.72	N/A	0.03±0.24	N/A	0.03±0.26	N/A

The values are expressed in Dioptres and represent average ± 1 standard deviation.

**M:** Spherical equivalent, **J0** and **J45:** Cylindrical vectorial components.

**VX:** Eye Refract, **WA:** WaveAnalyzer 700, **NIII:** TONOREF™ III, **NH:** Handheld ref/keratometer HandyRef-K, **PO:** PlusoptiX A12C, **SN:** NVision-K 5001, **SR:** Subjective refraction.

**Fig 1:**
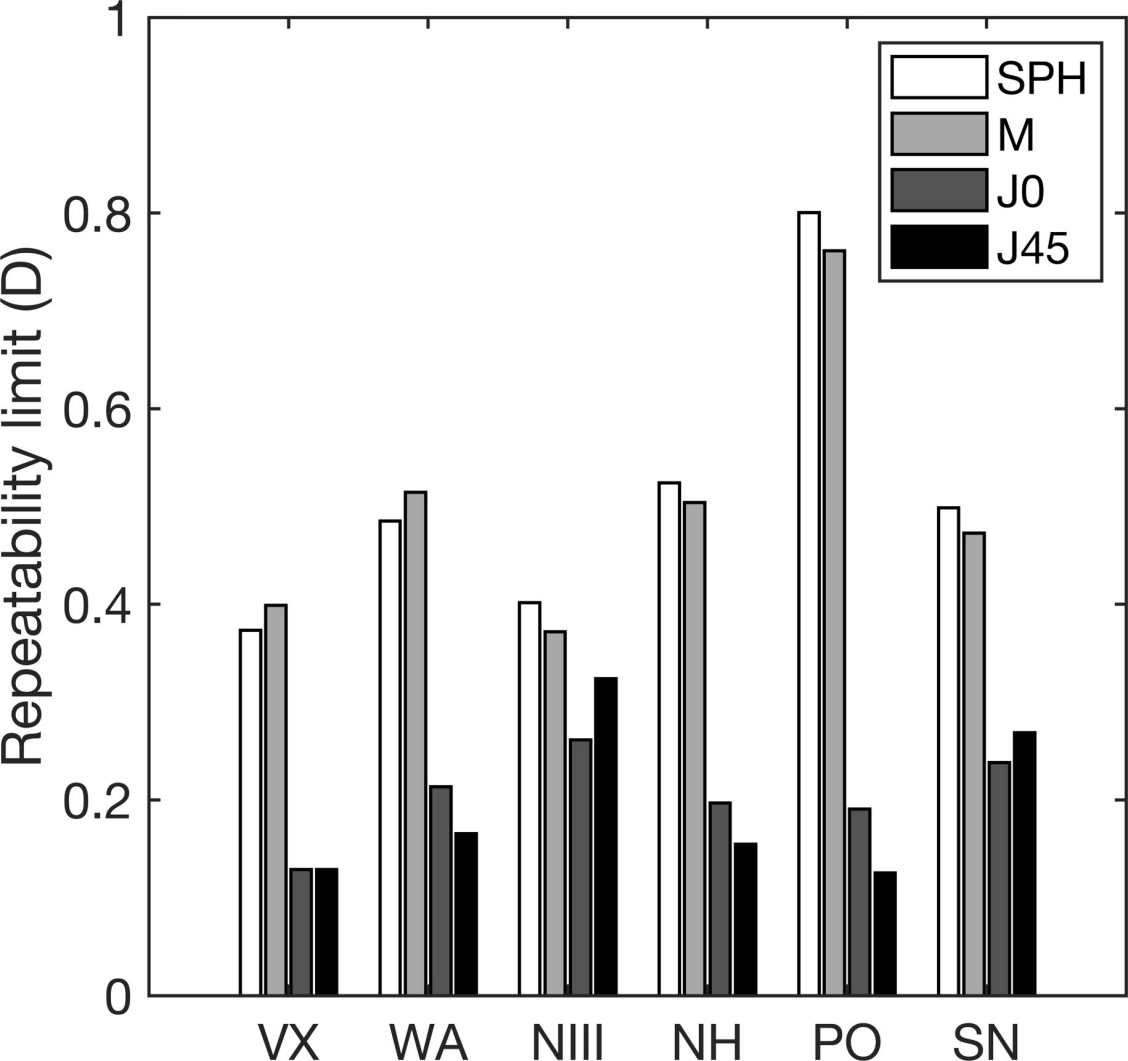
Repeatability limit for the measurements of sphere, spherical equivalent and the cylindrical vector components. SPH: Sphere, M: Spherical equivalent, J0 and J45: cylindrical vector components, VX: Eye Refract, WA: WaveAnalyzer 700, NIII: TONOREF™ III, NH: Handheld ref/keratometer HandyRef-K, PO: PlusoptiX A12C, SN:NVision-K 5001.

Fig 1 shows the repeatability limit of each autorefractor for the Sphere, M, and astigmatic components (J0, and J45). This figure shows that the differences in the repeatability limits between the sphere and M, and between J0 and J45 were minimal for all instruments (in all cases, the maximum difference was 0.07D). The repeatability limit values of the sphere and M were at least double than the values obtained for the astigmatic components for all autorefractors, except for the NIII where J0 and J45 were only 1.33 times lower.

The repeatability limit for the sphere and M were lower than 0.55D for all autorefractors but the PO, where the values were 0.80 and 0.75D, respectively. Regarding J0 and J45, the repeatability limits were lower than 0.35 for all AR.

### 3.2 Agreement between the subjective and objective refraction

**Fig 2:**
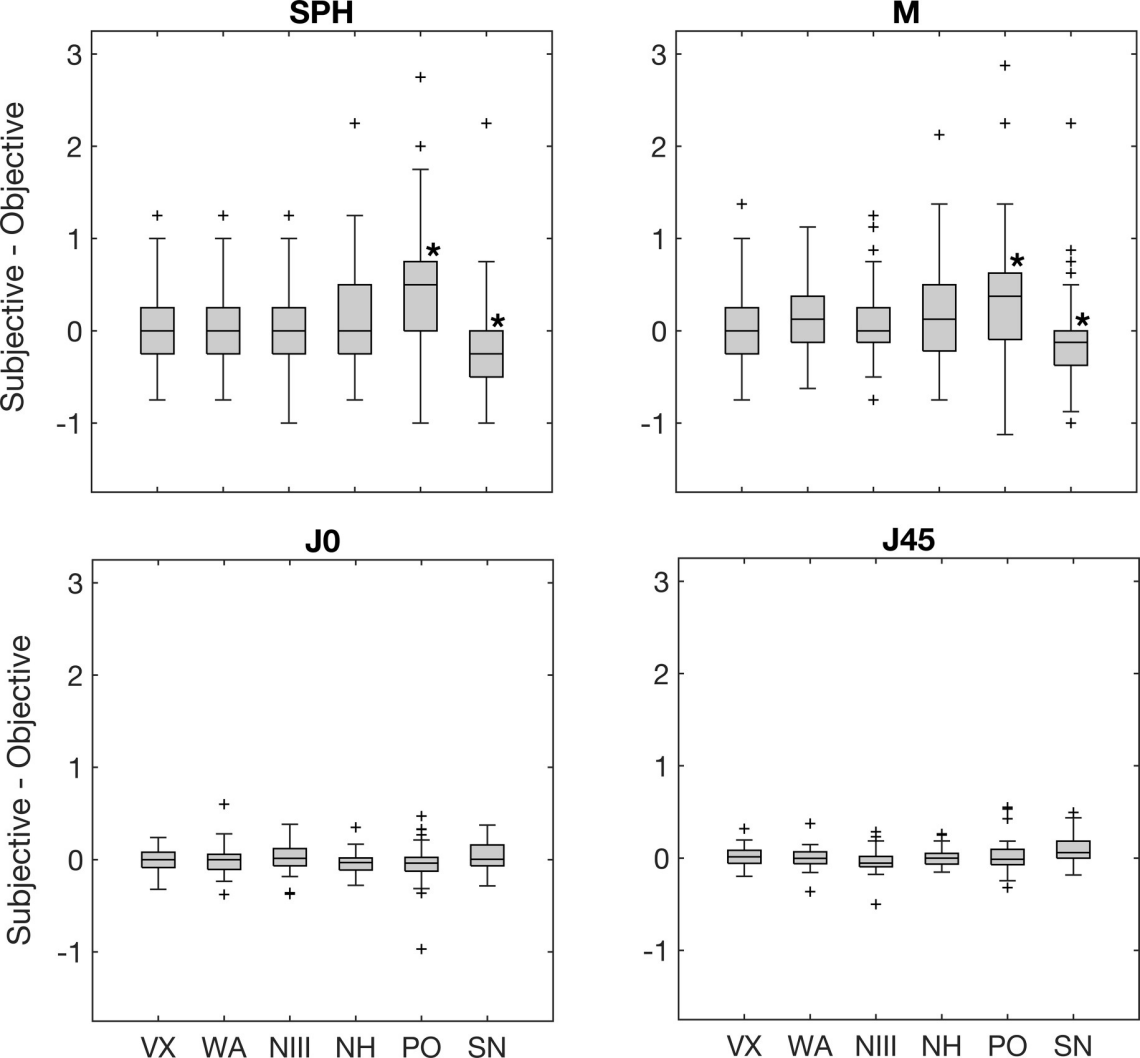
Average differences between subjective refraction and objective refraction for sphere, spherical equivalent and the cylindrical vector components. The asterisks (*) indicate statistically significant differences. The error bars represent the standard deviation of the differences. Check Fig 1 captions for abbreviations.

The differences between the subjective refraction and objective refraction for the sphere, M, J0, and J45 are shown in the box plot in Fig 2. In this figure, values larger than zero mean that the subjective refraction was less myopic or more hyperopic than the objective refraction, and vice versa. Also, the asterisks included in this figure indicate that the differences between the subjective refraction and autorefractor are statistically significant.

All tabletop autorefractors that include fogging (VX, WA, NIII) during the measurement provided the most similar values for sphere and M. Meanwhile, the tabletop autorefractor without fogging (SH) measured more negative values than the subjective refraction. Between both handheld autorefractors, the NH showed more similar values to subjective refraction than the PO, which measured less hyperopic (or more myopic) values than the subjective refraction. The differences between the subjective refraction and PO (p<0.001) and SN (p = 0.02) for sphere and M were statistically significant.

Regarding both astigmatic components, the differences with subjective refraction were minimal for all autorefractors. The only significant difference was obtained for the J45 between the subjective refraction and SN (p = 0.003).

**Fig 3:**
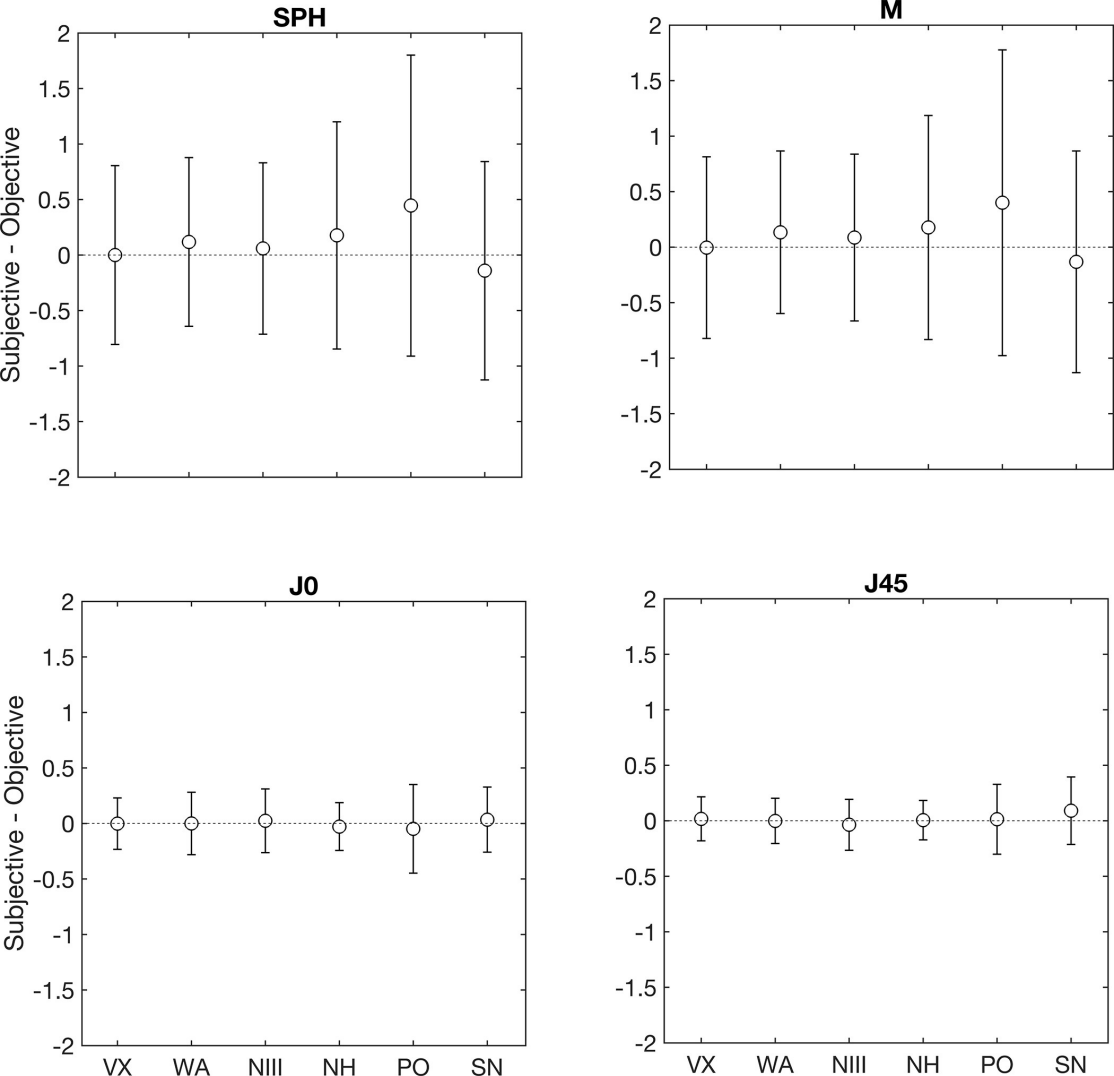
Bland-Altman plots for sphere, spherical equivalent and the cylindrical vector components. The open circle represents the mean difference between the subjective refraction and each autorefractor, and the error bars denote the 95% limits of agreement. Check Fig 1 captions for abbreviations.

Fig 3 summarises the results from the Bland-Altman plots for the sphere and each power vector component. On each graph, the white circle represents the mean difference between the subjective refraction and each autorefractor, and the error bars denote the 95% limits of agreement. As this figure shows, the mean difference for the sphere and M was lower than ±0.25D for each autorefractors except for the PO, where the value was +0.45D. The WA and PO resulted with the narrowest and widest limit of agreement intervals (1.50D, and 2.60D), respectively. For J0 and J45, the mean difference was lower than 0.10D and the interval of the limits of agreement ranged between 0.3 and 0.6D for all autorefractors.

## 4. Discussion

We evaluated the precision and accuracy of six different autorefractors that have different designs and measurement principles. Though the objective refraction measurements by the autorefractors do not consider the visual performance of the subjects and work mainly based on the optical quality of the eye, it will be interesting to know which of the autorefractor provides values that are closer to the subjective refraction. The repeatability of all autorefractors for measuring the spherical and M components was within 1.00D, and for the cylindrical components it was below 0.35D. Compared to subjective refraction, the mean difference for spherical and M components were on average below 0.50D for all autorefractors. However, the interval of the limit of agreement varied between 1.50 to 2.75D. Regarding the cylindrical components, the mean difference was close to zero, and the interval of the limit of agreement was also below 0.80D.

### 4.1 Effect of measurement principle

The two autorefractor (VX and NIII) that had the best precision and accuracy have different measurement principles (VX: wavefront and NIII: retinal image size). There were other autorefractors with the same measurement principle but showing worser repeatability and agreement values. As discussed below, the design of the autorefractor might play a bigger role in determining the measurement precision and accuracy.

### 4.2 Effect of fogging during the objective refraction measurements

Among the autorefractors that use fogging during the measurement, VX and NIII had better repeatability for the spherical and M components than the WA and NH. However, the differences in the repeatability for these autorefractors were less than 0.25D. Regarding the agreement with subjective refraction, the autorefractors that used fogging had the best agreement, except for the NH. The main difference between the NH and the others is that this is a handheld instrument, and the others are tabletop. The autorefractors without fogging (PO, and SN) had in general larger repeatability values and larger intervals of limits of agreement compared to the autorefractors that used fogging. A previous study has also shown that the use of additional fogging lenses while measuring refraction with autorefractors without inbuilt fogging is an effective way to control unwanted accommodative responses [21]. The effect of fogging on controlling accommodation and providing closer values to subjective refraction has been reported in other studies also [22–24]. The agreement among the fogging based autorefractors is also shown to be good in general [25].

### 4.3 Effect of open view during the objective refraction measurements

Among the open view autorefractors, the VX which also employs fogging showed the better repeatability and agreement than the other two (PO and SN). The open view autorefractor are known to control accommodation and provide reliable refraction measurements [26–28]. In a previous study comparing fogging-based open and closed view methods reported better agreement with the open view autorefractor [29]. Our results also show that the precision and accuracy of the open field autorefractors can be optimized by combining the fogging technique. One of the open field autorefractor with fogging also performed simultaneous binocular measurements (VX) which showed good repeatability and agreement. However, one of the closed field autorefractor with fogging also showed similar repeatability and agreement. This suggests that both with open and closed view instruments, the presence of fogging is more crucial. This again points out that the fogging used during the objective refraction measurements is important. All the closed view autorefractor used in this study employed fogging during the measurements. Among these, the tabletop autorefractors (WA and NIII) showed relatively better precision and accuracy compared to the handheld autorefractor (NH).

### 4.4 Effect of handheld design

The handheld autorefractors are mainly used in children [12, 15, 30, 31]. We evaluated two handheld autorefractors, one uses fogging (NH) and the other autorefractor that is based on photorefraction does not use fogging (PO). Between these two autorefractors, the one with fogging had better repeatability and agreement compared to the one without fogging. Previous studies have shown that the PO underestimates hyperopia in children [30, 31]. In the present study, we see a similar trend even among the adult subjects.

Both handheld autorefractors used in this study showed larger repeatability values and intervals limits of agreement compared to all the tabletop autorefractometers except SN. The head position and rotation could also play a major role in the precision and accuracy of these handheld autorefractors, as head tilt during measurements can affect the cylindrical measurements. In our study, the PO had the largest interval limit of agreement for the cylindrical components. On the contrary, the NH showed better agreement for the cylindrical components compared to the PO. This could be because of the differences in the head position, rotation, and fixation as NH had a head rest.

For 3 of the autorefractors (NIII, NH and SN) used in this study, the refraction measurement reported is the average of consists of 3 consecutive measurements in rapid succession. We performed this measurement and waited 2 minutes to repeat it again. The previous autorefractor precision studies have analyzed the repeatability with either 2 or 3 repeated measurements [10, 11, 30, 32, 33]. As a supplementary evaluation to assess how the repeatability varies with 3 repeated measurements, we evaluated one of the autorefractor (NIII) on a subset of 16 subjects. This is the autorefractor showed the best repeatability value for the spherical equivalent but not for the cylindrical components with the 2 repeated measurements (Fig 1). With 3 repeated measurements, the repeatability did not vary more than 0.1 D compared to 2 repeated measurements. The refractive error range measured in the present study is between -5.25 to +4.25D and the present results cannot be extrapolated to high refractive errors, especially in hyperopes. However, the majority of the clinical population in a regular optometry practice falls within the range included in this study.

The clinical accuracy of autorefractors is evaluated by comparing the measurements with the standard subjective refraction. In the present study, a wide range of autorefractor designs are compared with the subjective refraction values in order to elucidate the best autorefractor design. Based on the present findings, it can be concluded that inbuilt fogging is the most important feature followed by the open view in determining the precision and accuracy of the autorefractor values.

## Supporting information

S1 Data (XLSX)
